# Epidemiology and Outcomes of Ventilator-Associated Pneumonia in Saudi Arabian Intensive Care Units: A Systematic Review and Meta-Analysis

**DOI:** 10.3390/microorganisms14051145

**Published:** 2026-05-19

**Authors:** Abdullah A. Alshehri, Jehad A. Aldali, Ghina M. Alhuwaymani, Farah M. Alanazi, Yara K. Alsarhan, Shahad A. Almutairi, Abrar A. Altayeb

**Affiliations:** 1 Department of Clinical Pharmacy, College of Pharmacy, Taif University, Taif 21944, Saudi Arabia; 2 Department of Pathology, College of Medicine, Imam Mohammad Ibn Saud Islamic University (IMSIU), Riyadh 13317, Saudi Arabia; 3 College of Medicine, Imam Mohammad Ibn Saud Islamic University (IMSIU), Riyadh 13317, Saudi Arabia; 4 College of Medicine, Princess Nourah bint Abdulrahman University, Riyadh 13317, Saudi Arabia

**Keywords:** ventilator-associated pneumonia, intensive care unit, antimicrobial resistance, Gram-negative bacteria, *Acinetobacter baumannii*, *Klebsiella pneumoniae*, infection prevention, ventilator care bundle, epidemiology, systematic review, meta-analysis, Saudi Arabia

## Abstract

Ventilator-associated pneumonia (VAP) remains a major healthcare-associated infection in intensive care units (ICUs) and is associated with prolonged hospitalization, increased antimicrobial use, and high mortality. In Saudi Arabia, evidence on VAP epidemiology, microbiology, and outcomes is fragmented across settings. This study aimed to systematically review and synthesise the available evidence on VAP in Saudi Arabian ICUs. This study followed PRISMA guidelines and was prospectively registered in PROSPERO (CRD420261332740). A systematic search of PubMed, MEDLINE, Embase, and Web of Science was conducted for studies published between 2015 and 2025. Studies from Saudi Arabia reporting VAP incidence in ICUs were included. A random-effects model was used to pool incidence per 1000 ventilator-days. Risk of bias was assessed using Joanna Briggs Institute tools. Analyses were performed using R. Seven studies involving a total of 15,844 patients, representing multicentre studies, national surveillance data, and single-centre cohorts across diverse ICU settings. The pooled incidence of VAP was 8.50 episodes per 1000 ventilator-days (95% CI: 3.23–13.78), with substantial heterogeneity (I^2^ = 97%). Subgroup analysis showed higher incidence during baseline phases (12.46 per 1000 ventilator-days) compared with intervention phases (8.06 per 1000 ventilator-days), while surveillance estimates were lower. Gram-negative pathogens predominated, particularly *Acinetobacter baumannii*, often exhibiting multidrug resistance. VAP was associated with prolonged ICU stay, delayed extubation, and high mortality. Implementation of infection prevention bundles was associated with reductions in VAP incidence. Ventilator-associated pneumonia remains a significant burden in Saudi Arabian ICUs, characterised by substantial variability in incidence and a predominance of multidrug-resistant pathogens. Strengthening infection prevention measures, enhancing antimicrobial stewardship, and improving national surveillance systems are essential to reduce VAP burden and improve patient outcomes.

## 1. Introduction

Ventilator-associated pneumonia (VAP) remains one of the most significant healthcare-associated infections in intensive care units (ICUs), particularly among patients requiring invasive mechanical ventilation for more than 48 h. It is associated with prolonged ventilation, longer ICU and hospital stay, increased antimicrobial use, and substantial mortality and cost burdens. Despite advances in critical care and infection prevention, including ventilator care bundles, VAP remains a persistent challenge. This is driven by diagnostic uncertainty, variation in surveillance definitions, and the rising burden of multidrug-resistant organisms [1,2]. Beyond its clinical impact, VAP is also an important indicator of ICU quality and patient safety.

Globally, VAP burden varies across regions, hospital systems, and case definitions. Incidence is generally higher in low- and middle-income settings than in high-income countries. These differences reflect variation in infection control practices, staffing, antimicrobial stewardship, and surveillance systems. A systematic review in Asia reported substantial variation in incidence and pathogen profiles, highlighting the need for region-specific evidence [3]. Recent studies confirm that VAP is associated with worse outcomes, including prolonged ICU stay and delayed weaning from mechanical ventilation [4,5].

The epidemiology of VAP in Saudi Arabia warrants focused evaluation. The healthcare system is rapidly evolving, with expanding critical care capacity and increasing patient complexity. National efforts have aimed to improve patient safety and infection prevention. However, available data suggest variability in ICU-acquired infections, including VAP, across regions and institutions. Surveillance data from Ministry of Health hospitals and multicentre Gulf studies indicate that VAP remains a concern, despite improvements in some centres following prevention programmes [6,7]. Single-centre studies also show variation in incidence, microbiology, and outcomes across adult and paediatric ICUs, likely influenced by local factors such as case mix, staffing, and adherence to preventive measures [8,9].

Antimicrobial resistance is an additional challenge. VAP management requires early empiric antibiotics, but inappropriate or prolonged use may promote resistance and worsen outcomes. Studies from Saudi hospitals report a high burden of resistant Gram-negative pathogens in device-associated infections, emphasising the need for surveillance and stewardship-guided therapy [10]. This aligns with global concerns from the World Health Organization, which identifies antimicrobial resistance as a major threat to effective treatment and patient outcomes [11]. In VAP, these pressures highlight the need for accurate epidemiological evidence to guide prevention, diagnosis, and treatment in local contexts rather than relying on data from dissimilar settings.

Evidence on VAP in Saudi Arabia remains fragmented and inconsistent across regions, ICU settings, and study designs [12,13]. While some studies report reductions in incidence following implementation of ventilator care bundles, others suggest that effectiveness varies by adherence and local context. In addition, existing studies differ in reporting of incidence, microbiological patterns, antimicrobial resistance, and clinical outcomes, limiting comparability and reliable national estimates. These gaps highlight the need for a comprehensive synthesis of available evidence. Therefore, this study aimed to systematically review and quantitatively synthesise data on VAP in Saudi Arabian ICUs, including incidence, microbiological profile, antimicrobial resistance patterns, clinical outcomes, and the impact of infection prevention interventions.

## 2. Methods

### 2.1. Study Design and Protocol Registration

This systematic review and meta-analysis were conducted in accordance with the Preferred Reporting Items for Systematic Reviews and Meta-Analyses (PRISMA) guidelines [14,15]. The study protocol was prospectively registered in the International Prospective Register of Systematic Reviews (PROSPERO) under registration number CRD420261332740.

### 2.2. Search Strategy and Eligibility Criteria

A comprehensive literature search was performed to identify relevant studies published between January 2015 and December 2025. Electronic databases searched included PubMed, MEDLINE, Embase and Web of Science. The search strategy combined keywords and controlled vocabulary related to VAP and Saudi Arabia. Core search terms included combinations of “ventilator-associated pneumonia” OR “VAP” AND “intensive care unit” OR “ICU” AND “Saudi Arabia”. Reference lists of included studies and relevant review articles were also screened to identify additional eligible studies through backward citation searching.

Studies were eligible for inclusion if they were conducted in Saudi Arabia, involved patients admitted to ICUs, and reported data on VAP. Only peer-reviewed studies published in English between 2015 and 2025 were included. Eligibility was based on publication date; therefore, studies with data collection periods prior to 2015 were included if they were published within the specified timeframe. Studies were excluded if they were case reports, conference abstracts, editorials, letters, or review articles, lacked ICU-level VAP incidence or outcome data, reported ventilator-associated events (VAEs) or other healthcare-associated infections without VAP-specific data, focused solely on microbiological pathogen analyses without epidemiological denominators, or represented duplicate publications.

### 2.3. Study Selection and Data Extraction

All records identified through database searches were imported into reference management software and duplicates were removed. Titles and abstracts were screened to identify potentially relevant studies. Full-text articles of eligible studies were retrieved and assessed against the predefined inclusion and exclusion criteria. When the full text of potentially eligible articles was unavailable, the corresponding authors were contacted to request access to the complete manuscript. However, no response was received after two attempts.

Data from included studies were extracted using a structured data extraction form developed for this review. Extracted information included study identification details such as author name and year of publication, study location and hospital setting, ICU type, study design, and study duration. Additional information collected included the number of patients included, number of ventilated patients, number of VAP cases, ventilator-days when available, diagnostic criteria used to define VAP, microbiological findings, antimicrobial resistance patterns, clinical outcomes associated with VAP, and reported infection prevention interventions. When studies reported multiple study periods, such as baseline and intervention phases, each period was extracted separately for the purpose of quantitative analysis.

### 2.4. Statistical Analysis

Studies reporting clinically and methodologically comparable VAP incidence density estimates per 1000 ventilator-days were eligible for quantitative meta-analysis. A random-effects meta-analysis model was used to pool incidence estimates due to anticipated clinical and methodological heterogeneity across studies, including differences in ICU populations, study designs, and infection prevention strategies. When studies reported separate baseline and intervention periods, these estimates were treated as independent observations in the meta-analysis. Statistical heterogeneity was assessed using Cochran’s Q test and the I^2^ statistic.

All analyses were conducted using R (R Foundation for Statistical Computing, Vienna, Austria), with meta-analysis packages. Pooled estimates were presented with 95% confidence intervals and visualised using forest plots.

Studies that did not report ventilator-day denominators were not eligible for quantitative pooling and were therefore included in the narrative synthesis, which summarized VAP frequency, microbiological profiles, antimicrobial resistance patterns, clinical outcomes, and infection prevention interventions.

### 2.5. Risk of Bias Assessment

The methodological quality of the included studies was assessed using the Joanna Briggs Institute (JBI) critical appraisal tools appropriate for each study design. Separate JBI checklists were applied for cohort studies, cross-sectional studies, and surveillance-based analyses. The assessment evaluated several methodological domains, including similarity of study groups, validity and reliability of exposure measurement, identification and management of confounding factors, outcome measurement, adequacy of follow-up, and appropriateness of statistical analysis. Each item was rated as “yes,” “no,” “unclear,” or “not applicable” according to JBI guidance. The overall risk of bias for each study was judged as low, moderate, or high based on the number and severity of concerns across JBI domains.

## 3. Results

### 3.1. Study Selection

**Figure 1:**
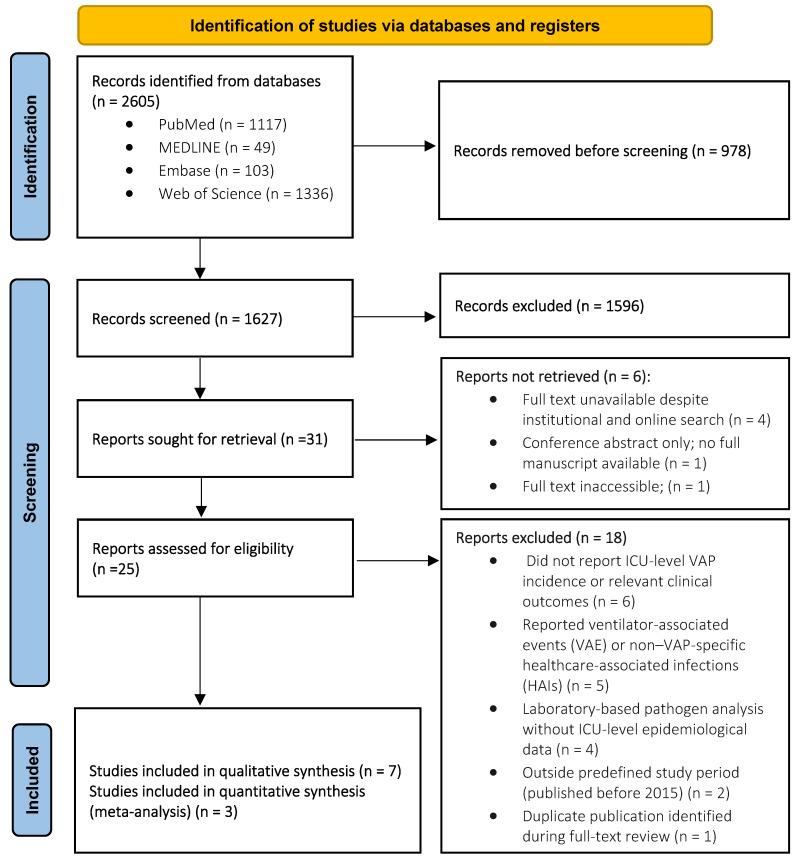
PRISMA 2020 flow diagram of study selection for the systematic review and meta-analysis.

The database search identified 2605 records. After removal of 978 duplicates, 1627 titles and abstracts were screened. Subsequently, 31 full-text articles were sought for retrieval. Six reports were not retrieved, and 25 full-text articles were assessed for eligibility. Of these, 18 studies were excluded based on predefined criteria. Ultimately, seven studies were included in the qualitative synthesis, of which three studies met the criteria for quantitative meta-analysis (Figure 1).

### 3.2. Characteristics of Included Studies

The seven included studies were conducted across several regions in Saudi Arabia, including Jeddah, Al-Ahsa, Albaha, and Aljouf, as well as multicenter and national surveillance studies involving multiple hospitals and ICUs. The studies were performed in different ICU settings, including adult ICUs, pediatric ICUs (PICU), and mixed ICUs. Regarding study design, the included studies comprised one prospective before–after cohort study, two retrospective cohort studies, one retrospective cross-sectional study, and two surveillance-based studies. The data collection periods of the included studies ranged from 2013 to 2023, with durations varying from one to four years.

Among the studies that reported sample size, a total of 15,844 patients were included. Several studies reported VAP incidence as episodes per 1000 ventilator-days, with rates ranging from 1.17 to 18 episodes per 1000 ventilator-days across different ICU settings. The detailed characteristics of the included studies are presented in Table 1.

### 3.3. Incidence of Ventilator-Associated Pneumonia

#### Meta-Analysis of VAP Incidence

**Figure 2:**
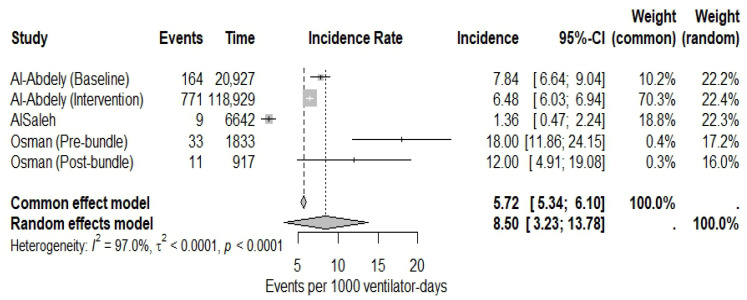
Forest plot of the multi-level meta-analysis of ventilator-associated pneumonia incidence per 1000 ventilator-days across included studies.

Four studies reported VAP incidence as episodes per 1000 ventilator-days; however, three studies provided clinically and methodologically comparable estimates and were included in the meta-analysis [16,17,21]. Consistent with the predefined eligibility criteria for quantitative synthesis, one study was included in the narrative synthesis but excluded from the meta-analysis due to lack of comparability [19]. Because two studies reported separate baseline/intervention phases, five incidence estimates were included in the quantitative synthesis. Using a random-effects model, the pooled VAP incidence was 8.50 episodes per 1000 ventilator-days (95% CI: 3.23–13.78). Substantial heterogeneity was observed among the included estimates (I^2^ = 97.0%, *p* < 0.0001). The forest plot of the multi-level meta-analysis is shown in Figure 2.

**Figure 3:**
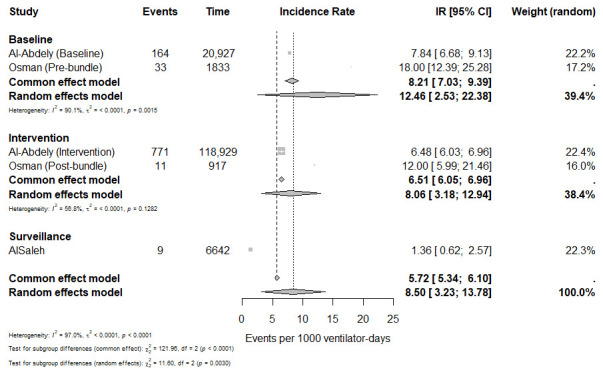
Subgroup meta-analysis of ventilator-associated pneumonia incidence per 1000 ventilator-days stratified by study phase (baseline, intervention, and surveillance).

A subgroup meta-analysis according to study phase demonstrated higher incidence during baseline phases (12.46 episodes per 1000 ventilator-days; 95% CI: 2.53–22.38) compared with intervention phases (8.06 episodes per 1000 ventilator-days; 95% CI: 3.18–12.94). The surveillance estimate reported the lowest incidence (1.36 episodes per 1000 ventilator-days; 95% CI: 0.47–2.24). The difference between subgroups was statistically significant (*p* = 0.003) (Figure 3).

Studies that did not report ventilator-day denominators were not included in the meta-analysis. In one cohort study, 33 of 367 mechanically ventilated patients (8.99%) developed VAP [5]. Another study reported a markedly high proportion of VAP cases (93%) among ventilated ICU patients with multidrug-resistant *Acinetobacter baumannii* respiratory infections [20]. In addition, 14 VAP cases were identified among ICU patients with healthcare-associated infections, although ventilator-day denominators were not available to calculate standardized incidence density [18]. A national surveillance report documented 184 VAP events with an incidence of 1.53 per 1000 ventilator-days, although the estimates primarily reflected pediatric and neonatal ICUs due to changes in surveillance practices for adult ICUs [19].

### 3.4. Microbiological Profile and Antimicrobial Resistance

Gram-negative pathogens predominated in the studies reporting microbiological data. The most frequently identified organisms were *Acinetobacter baumannii, Klebsiella pneumoniae*, and *Pseudomonas aeruginosa*. *A. baumannii* was the leading pathogen in several adult ICU studies and was frequently associated with multidrug-resistant or extensively drug-resistant phenotypes. Carbapenem resistance was reported in multiple cohorts, particularly among *A. baumannii* isolates. Detailed pathogen distribution and antimicrobial resistance patterns are summarized in Table 2.

### 3.5. Clinical Outcomes and Infection Prevention Interventions

Clinical outcomes associated with VAP were variably reported across the included studies. Mortality outcomes were reported in several cohorts. In one cohort study, 28-day mortality among patients with VAP reached 36.36%, although the difference compared with non-VAP patients was not statistically significant [5]. Another study examining ventilated ICU patients with multidrug-resistant *Acinetobacter baumannii* infections reported an overall mortality rate of 74%, with higher mortality observed among patients with COVID-19 [20]. In a pediatric ICU study evaluating a VAP prevention bundle, VAP-attributable mortality decreased from approximately 6% before implementation to 3% after implementation (Osman, 2020) [21].

Several studies also reported indicators of disease severity and resource utilization. Patients with VAP experienced longer ICU length of stay (median 19.5 vs. 13 days) and prolonged time to extubation (median 13.5 vs. 6 days) compared with patients without VAP [5]. In the same study, chronic ventilation occurred in 36.36% of VAP patients compared with 19.76% of non-VAP patients, while extubation failure was more frequent among patients with VAP (24.24% vs. 14.48%), although this difference was not statistically significant.

Infection prevention interventions were evaluated in two studies. A large multicenter study involving 37 adult ICUs across 22 hospitals demonstrated that implementation of a multidimensional infection control program was associated with a reduction in VAP incidence from 7.84 to 4.74 episodes per 1000 ventilator-days, representing a 39% reduction in VAP rates [16]. Similarly, implementation of a VAP prevention bundle in a pediatric ICU was associated with a decline in VAP incidence from 18 to 12 episodes per 1000 ventilator-days following bundle implementation [21].

### 3.6. Risk of Bias Assessment 

Due to differences in study design, risk-of-bias domains varied across studies. However, most studies demonstrated low to moderate methodological quality overall. Key domains, including population selection, exposure measurement, outcome assessment, and statistical analysis, were generally rated as low risk. Confounding was the main source of bias, as several studies did not clearly identify or address potential confounders. In addition, information on follow-up completeness was inconsistently reported in some cohort studies. Despite these limitations, all studies employed appropriate statistical analyses for the reported outcomes. A detailed summary of the risk-of-bias assessment is presented in Table S1.

## 4. Discussion

This systematic review and meta-analysis evaluates the available evidence on VAP in ICUs across Saudi Arabia. Seven studies were included, representing multiple ICU settings, regions and study designs. Overall, VAP incidence demonstrated a moderate but highly variable burden across studies. Considerable heterogeneity was observed, likely reflecting differences in ICU populations, surveillance practices, diagnostic criteria, and infection prevention strategies, as well as variation in incidence reporting methods. Higher incidence during baseline phases compared with intervention periods suggests a measurable impact of infection prevention interventions. These findings should therefore be interpreted cautiously given the substantial methodological and clinical heterogeneity across included studies. Across studies, VAP was consistently associated with adverse clinical outcomes, including prolonged ICU stay, delayed extubation, and increased mortality.

The pooled VAP incidence observed in this review falls within the range reported globally but appears higher than rates reported in many high-income healthcare systems. Studies from North America and Western Europe have reported VAP incidence densities typically ranging between 1–5 episodes per 1000 ventilator-days, particularly in ICUs with established surveillance systems and standardized prevention bundles. By contrast, studies from several middle-income countries and parts of Asia, Latin America, and the Middle East have reported higher incidence rates, frequently exceeding 10 episodes per 1000 ventilator-days [22]. The substantial heterogeneity identified in this analysis likely reflects differences in ICU case mix, diagnostic criteria, surveillance definitions, and adherence to infection prevention protocols across institutions. Additional contributors may include variation in ICU type (adult, mixed, and paediatric ICUs), differences in study design and surveillance methodology, regional variation in antimicrobial resistance patterns, and inconsistent reporting of ventilator-day denominators and microbiological data across studies.

The predominance of Gram-negative pathogens, particularly A. baumannii and K. pneumoniae, aligns with findings from regional and global surveillance studies of VAP epidemiology. These pathogens are well recognised for their ability to develop multidrug resistance and persist in hospital environments, particularly in critical care settings. Carbapenem resistance among A. baumannii has been increasingly reported in several countries in the Middle East and Asia and represents a major challenge for antimicrobial stewardship and infection control [23,24,25].

The reduction in VAP incidence following implementation of infection prevention bundles aligns with evidence from international studies demonstrating the effectiveness of multidimensional strategies, including ventilator care bundles, staff education, surveillance feedback, and antimicrobial stewardship [26,27,28,29,30]. These findings support the continued implementation and scaling of evidence-based infection prevention interventions in ICU settings.

These findings have important implications for clinical practice and health policy in Saudi Arabia and comparable healthcare systems. The marked variability in reported VAP incidence highlights the need for standardized national surveillance systems, unified diagnostic criteria, and consistent reporting frameworks to improve comparability across institutions and enable more accurate monitoring of infection trends. Establishing robust surveillance infrastructure would also facilitate benchmarking and support targeted quality improvement initiatives at the institutional and national levels. Moreover, the predominance of multidrug-resistant Gram-negative pathogens further underscores the urgency of strengthening antimicrobial stewardship programmes and infection control practices in ICUs. Optimising empiric antibiotic selection based on local epidemiology, promoting de-escalation strategies, and integrating microbiological surveillance into clinical decision-making are critical to limit resistance and improve outcomes. At the clinical level, consistent implementation and monitoring of evidence-based interventions—such as ventilator care bundles, strict adherence to hand hygiene and infection prevention protocols, elevation of the head of the bed, sedation minimisation, and early weaning from mechanical ventilation—may substantially reduce VAP incidence. Embedding these practices within multidisciplinary care models and ensuring staff training and compliance auditing are essential to sustain their effectiveness. Collectively, these strategies may help reduce VAP burden, improve patient outcomes, and optimise resource utilisation in ICU settings.

To our knowledge, this is the first systematic review and meta-analysis to comprehensively assess the epidemiology of VAP in Saudi Arabian ICUs. Strengths of this study include a comprehensive search strategy across multiple databases, adherence to PRISMA guidelines, and the integration of quantitative meta-analysis with narrative synthesis, allowing for a broad evaluation of incidence, microbiology, antimicrobial resistance, clinical outcomes, and prevention strategies across diverse ICU settings. However, several limitations should be considered. Only a limited number of studies reported ventilator-day denominators required for calculating incidence density, which reduced the number of studies eligible for quantitative pooling. Substantial heterogeneity was observed across studies, likely reflecting differences in ICU case mix, surveillance practices, diagnostic criteria, and infection prevention strategies. Additionally, microbiological and antimicrobial resistance data were incompletely and inconsistently reported across studies, limiting detailed synthesis of resistance rates, resistance classes, and resistance gene profiles [31,32]. Variability in surveillance practices and ICU management approaches may have further affected comparability across studies.

## 5. Conclusions

Ventilator-associated pneumonia remains a significant complication among mechanically ventilated patients in Saudi Arabian ICUs, with substantial variability in incidence across studies. Gram-negative pathogens, particularly *Acinetobacter baumannii* and *Klebsiella pneumoniae*, predominated and were frequently associated with multidrug resistance. VAP was associated with prolonged ICU stay, delayed extubation, and increased mortality. Infection prevention bundles were associated with reductions in VAP incidence. However, interpretation of these findings should consider the methodological heterogeneity and limited comparability across included studies. Strengthening infection prevention measures, antimicrobial stewardship, and surveillance systems remains essential to reduce VAP burden and improve patient outcomes.

## Figures and Tables

**Table 1:** Characteristics of the included studies investigating ventilator-associated pneumonia in intensive care units in Saudi Arabia.

Study	Region	ICU Type	Study Design	Study Period	Sample Size (Patients)	Age (Mean ± SD)	Male (%)	Ventilator Days	VAP Cases	VAP Incidence (per 1000 MV-days)
Al-Abdely 2018 [16]	Multicenter, (37 ICUs)	Adult ICU	Prospective before–after cohort	Sep 2013–Feb 2017	Baseline: 2261	44.2 ± 22.7	67.6	20,927	164	7.84
					Intervention: 12,700	43.0 ± 26.1	67.0	118,929	771	4.74
AlSaleh 2023 [17]	Al-Ahsa	Mixed ICU	Surveillance (retrospective)	Jan–Dec 2022	NR	NR	NR	6642	9	1.17
Alshammari 2025 [18]	Aljouf	Mixed ICU	Retrospective cross-sectional	Jan 2020–Dec 2023	260	NR	56.5	NR	14	NR
Alsheddi 2023 [19]	National (106 hospitals)	Mixed ICU	National surveillance	Jan–Dec 2022	NR	NR	NR	120,359	184	1.53
Hafiz 2023 [20]	Albaha	Adult ICU	Retrospective study	Mar 2020–Mar 2022	115	64.9 ± 19.7 *	66	NR	107	NR
Osman 2020 [21]	Jeddah	Paediatric ICU	Cohort (before–after bundle)	Jan 2015–Mar 2018	Pre-bundle: 95	NR	56	1833 **	33	18
					Post-bundle: 36	NR	44	917 **	11	12
Turkistani 2024 [5]	Jeddah	Mixed ICU	Retrospective cohort	Jun 2016–May 2020	367	Median: VAP 45 (0.08–89); Non-VAP 48 (0.08–96)	63	NR	33	NR

* Overall mean age calculated from sex-specific data reported in the original study. ** Ventilator-days were derived from the reported VAP incidence and number of VAP cases. NR = Not reported.

**Table 2:** Microbiological profile and antimicrobial resistance characteristics of ventilator-associated pneumonia across the included studies.

Study (Year)	Most Common Pathogen	Second Pathogen	MDR/XDR Reported	Carbapenem Resistance
Al-Abdely (2018) [16]	*Acinetobacter baumannii*	*Klebsiella pneumoniae*	Yes	Not specified
AlSaleh (2023) [17]	NR *	NR *	NR *	NR *
Alshammari (2025) [18]	*Klebsiella pneumoniae*	*Acinetobacter baumannii*	Not reported	Not reported
Alsheddi (2023) [19]	NR *	NR *	NR *	NR *
Hafiz (2023) [20]	*Acinetobacter baumannii*	NR	Yes	High resistance reported
Osman (2020) [21]	*Pseudomonas aeruginosa*	*Klebsiella pneumoniae*	Not reported	Reported
Turkistani (2024) [5]	*Klebsiella pneumoniae*	*Acinetobacter baumannii*	Yes	Not reported

* NR: Not reported. Some included studies did not provide microbiological or antimicrobial resistance data.

## Data Availability

The raw data supporting the conclusions of this article will be made available by the authors on request.

## Supplementary Materials

The following supporting information can be downloaded at: https://www.mdpi.com/article/10.3390/microorganisms14051145/s1, Table S1: Risk of bias assessment of included studies using Joanna Briggs Institute tools.
