# Supplementary material for: Epidemiology and Outcomes of Ventilator-Associated Pneumonia in Saudi Arabian Intensive Care Units: A Systematic Review and Meta-Analysis
Source: Microorganisms. 2026 May 19;14(5):1145. doi: 10.3390/microorganisms14051145 (PMC13209618; doi:10.3390/microorganisms14051145)
Supplement: Supplementary file 1 [file microorganisms-14-01145-s001.zip › microorganisms-4269132-Supplementary.pdf]

Table S1 Risk of bias assessment of included studies using Joanna Briggs Institute tools

Study (Author, Year)	Study Design	Selection Bias	Exposure Measurement	Confounding	Outcome Measurement	Follow-up	Statistical Analysis	Overall Risk
Al-Abdely, 2018 [16]	Cohort	Low	Low	Low	Low	Low	Low	Low
AlSaleh, 2023 [17]	Surveillance analysis	Low	Low	Low	Low	NA	Low	Low
Alshammari, 2025 [18]	Cross-sectional	Low	Unclear	Low	Low	NA	Low	Low
Alsheddi, 2023 [19]	Multicenter surveillance	Low	Low	Low	Low	NA	Low	Low
Hafiz, 2023 [19]	Retrospective cohort	Low	Low	Moderate	Low	Unclear	Low	Moderate
Osman, 2020 [20]	Cohort	Low	Low	Moderate	Low	Low	Low	Moderate
Turkistani, 2024 [5]	Retrospective cohort	Low	Low	Unclear	Low	Unclear	Low	Moderate

The included studies comprised different observational designs, the appropriate checklist was applied according to study design. Follow-up domains were not applicable to cross-sectional and surveillance studies.
